# The outcome of genetic and non-genetic pediatric cardiomyopathies

**DOI:** 10.1186/s43044-024-00473-7

**Published:** 2024-04-03

**Authors:** Ali AlAlakhfash, Luciano Agati, Giuseppe Mazzesi, Dalia Elhobi, Abdullah Alqwaiee, Khalid Alhory, Abdulrahman Almesned, Zuhair Alhasnan, Abdullah Alwadai

**Affiliations:** 1 Pediatric Cardiology Department, Prince Sultan Cardiac Center-Qassim, Qassim Health Cluster, MOH, P O BOX 896, 51421 Buraydah, Saudi Arabia; 2 Direttore U.O. “Diagnostica e Terapia Cardiovascolare”, Dipartimento di Scienze Cardiovascolari E Respiratorie, Cattedra Di Cardiologia, Università Sapienza Roma, Policlinico Umberto I, PadiglioneRome, Italy; 3 Department of General Surgery and Cardiothoracic Surgery, “Paride Stefanini”, Sapienza University of Rome, Rome, Italy; 4 KFSH-RC, Riyadh, Saudi Arabia

## Abstract

**Background:**

Pediatric cardiomyopathies (CMP) can be familial or idiopathic with increasing detection of genetic mutations. The study is a retrospective single-center review of cardiomyopathy patients from January 2011 to May 2020. Results of the genetic study, as well as the outcome, were reported. Patients were divided according to the type of CMP, age of presentation, and EF at presentation. Univariate and multivariate analysis and ROC and survival curves were done.

**Results:**

We reported 229 patients under 14 years of age with a diagnosis of cardiomyopathy, most commonly DCM (160 patients (70%)) followed by HCM (26.2%). 52% presented at 6 months of age or less and 119 (52%) required ICU admission at presentation. The genetic and or metabolic disorder was confirmed in 21.4% of patients, most commonly VLCAD defect (16, 7%) and ELAC2 gene defect (10, 4.4%). During the disease course, 88 patients (38.4%) died (48 with DCM, 39 with HCM, and 1 with RCM). An EF of 20% or less at presentation and presentation at 6 months of age or less carries a risk for mortality in patients with DCM and HCM, respectively (RR 3.88 and 2.06 and OR of 11.09 and 4.35, respectively). Death was more common among HCM patients especially patients with positive genetic abnormality compared with patients with DCM.

**Conclusions:**

The mortality for CMP in children reaches up to 40%, (30% in DCM and 65% in HCM patients). Mortality was higher in those with HCM, DCM with EF of 20% or less, and HCM presented at 6 months of age or less. Whole-exome and/or whole-genome sequencing is advised for all patients of CMP and at-risk family members.

## Background

Cardiomyopathies (CMP) represent a diverse range of myocardial disorders, displaying different structural and functional characteristics often linked to a genetic origin or cause [1].

CMP can occur at any age, and it is a common cause of heart failure and heart transplantation in children. Pediatric cardiomyopathies can result from coronary artery abnormalities, tachyarrhythmia, exposure to infection or toxins, or secondary to other underlying disorders. In pediatric patients, most cardiomyopathy patients are non-ischemic. The exact incidence of pediatric cardiomyopathy is unknown, especially in developing countries. In developed countries, the incidence of CMP is estimated to be around 4.8 per 100,000 infants and 1.3 per 100,000 children less than 10 years of age [2]. CMP can be classified into primary or acquired, or as familial/genetic and non-familial/non-genetic. According to the pathophysiology and characteristics of the myocardial muscle, CMP are typically categorized into four types. These include hypertrophic CMP (34.2%), dilated CMP (53.8%), restrictive CMP (3.2%), and other or mixed types of CMP (8.9%) [3].

Echocardiography is the first tool to diagnose cardiomyopathy. It can differentiate between the different types of CMP (dilated cardiomyopathy (DCM), hypertrophic cardiomyopathy (HCM), restrictive cardiomyopathy (RCM), and others) and for follow-up [4, 5].

Cardiac magnetic resonance imaging (CMRI) is also used frequently in patients with suspected or proven CMP. CMRI helps to diagnose as well as to give hemodynamic information and prognostic guidance [6].

The cause of CMP is unknown, or idiopathic in most patients. Pediatric patients presenting with cardiomyopathy need to be screened for certain metabolic and genetic disorders known to be associated with cardiomyopathy (e.g., carnitine deficiency, fatty acid oxidation disorders, organic acidemias, storage disorders, and congenital glycosylation disorders). Genetic causes of CMP, especially the hypertrophic type, were discovered, and new or novel genes are continuously discovered to be associated with HCM [7]. Inborn errors of metabolism are an important cause of cardiomyopathy in pediatrics, especially hypertrophic cardiomyopathy [8].

In addition to routine newborn screening tests, it is recommended to do genetic testing (whole-exome sequencing or whole-genome sequencing) for all patients with cardiomyopathy [9]. Genetic variants can be inherited, or occur de novo [10].

The study aimed to provide a comprehensive understanding of pediatric cardiomyopathy in a specific region and to identify potential predictors for poor outcomes. The research utilized data from a local hospital database to analyze the distribution of different types of pediatric cardiomyopathy, as well as factors such as age, sex, and potential causes of the condition. Furthermore, the research aimed to evaluate the outcomes associated with various cardiomyopathy types and identify factors predicting adverse outcomes, such as death or the necessity for transplantation.

The results of this study could contribute to a better understanding of pediatric cardiomyopathies in the studied geographic area, with potential implications for enhancing the diagnosis, treatment, and management of affected patients. Moreover, by identifying potential indicators of unfavorable outcomes, the research may help healthcare professionals in developing targeted interventions to improve patient outcomes. This content is included in a PhD thesis presented at Sapienza University of Rome—Italy.

## Methods

The study is a retrospective single-center review of all patients, 14 years or less, with a diagnosis of cardiomyopathy admitted and/or seen in the outpatient department during the period from January 2011 to May 2020. All patients with a diagnosis of cardiomyopathy and a follow-up period of 6 months or more and patients who died because of cardiomyopathy regardless of the follow-up period were included.

Patients excluded include:

1. Patients with cardiomyopathy associated with congenital heart diseases like critical aortic stenosis (AS), coarctation of the aorta (CoA), or anomalous left coronary artery from the pulmonary artery (ALCAPA)
2. Patient with depressed cardiac function post-cardiac surgery.
3. Patients with dilated ventricles due to volume overload associated with CHD.
4. Depressed cardiac function related to chronic arrhythmia, pulmonary parenchymal or vascular disease, and immunologic disease.
5. Infants of diabetic mothers.
6. Chemotherapy-associated cardiotoxicity.
7. Any patient with hypertrophy related to drugs known to cause hypertrophy.

In all patients, the final diagnosis and type of cardiomyopathy were made by an expert pediatric cardiologist(s) based on the clinical presentation, ECG, chest x-ray, and most importantly on the echocardiographic findings. Data were collected from the time of initial presentation till the last follow-up or death. The timing of follow-up depends on the age of the patient, the patient’s clinical condition, the degree of ventricular function impairment, the acuteness of the illness, and the family’s social background. Usually for recently diagnosed cases with CMP, we give near appointments (after 2 to 4 weeks of discharge) and then every 1 to 3 months until the patient’s condition stabilizes, and the medications are built up and well tolerated by the patient. In every visit, the patient will be assessed clinically and an ECG and transthoracic echocardiography will be performed. We had direct telephone contact with the families of patients who missed their appointments. Data collected include the demographic data, echocardiographic data, the type of CMP, clinical presentation, mode of presentation, admission on initial presentation, ICU admission and course, IVIG administration, medications which give results of the genetic and metabolic screening, recurrent hospital admissions, current patient condition and functional class, current medications and the outcome. The outcome could be an improvement of cardiac function, static cardiac condition, alive with symptoms, alive and asymptomatic, died or had heart transplantation. Unfavorable outcome was defined as the occurrence of either death or heart transplant at the last follow-up.

Improvement in cardiac function was defined as improvement in ventricular systolic function with an increase in the EF to 50% or more and a decrease in left ventricle end-diastolic dimensions (LVEDd) to a z-score of 2 or less for age, gender and body surface area.

Categorical variables are presented as numbers and percentages. Numerical variables are presented as mean ± standard deviation or medians with interquartile ranges, minimum, and maximum as appropriate.

The chi-square test was used to compare categorical variables (Fisher’s exact test was used when the expected frequency was less than 10) and Student’s t test (or Mann–Whitney U test for abnormally distributed data) was used to compare numerical variables. The ROC curves were created to predict the factor with the highest sensitivity and specificity for poor outcomes. Factors linked to unfavorable outcomes (lack of improvement, transplant, and/or mortality) were identified through multivariable analysis. The results were presented as relative risk and odds ratio (OR) with a 95% confidence interval. A *P*-value < 0.05 was deemed statistically significant. Statistical analysis was conducted using the IBM SPSS Statistics 25 program.

We divided patients according to (1) The different types of cardiomyopathy. (2) The EF at presentation (for DCM patients) into either EF 20% or less or more than 20%. (3) The age of presentation, into those presented at an age of 6 months or less or more than 6 months of age.

Group comparisons and ROC curve creation were conducted. We examined predictors for mortality according to the different types of cardiomyopathy, the age of presentation, the EF at presentation, the gender differences, the effect of the clinical condition on initial presentation, the effect of the management given during the acute and chronic course, requirement for ICU admission, effect of the different ICU management, IVIG administration impact on DCM course, effect of the genetic abnormalities, and the influence of chronic medications on long-term outcomes.

For the genetic investigation, a detailed consent was obtained from the family prior to collecting blood samples from the patient and both parents. Based on the clinical diagnosis and the likelihood of mutation, a genetic test was categorized as either a targeted genetic test (TGT) with a targeted mutation test, a single-gene test, or a multi-gene panel as for Noonan syndrome, or an untargeted genetic test with whole-exome sequencing (WES) or whole-genome sequencing (WGS). Several bioinformatics tools were used to filter the variants [11]. Data were handled anonymously, and approval for the study was granted by the hospital research committee.

Dilated cardiomyopathy (DCM) is identified when there is dilatation of the left ventricle (LV) along with impaired systolic function, without the presence of hypertension, coronary artery disease, valve issues, congenital heart disease, or other conditions causing volume overload [11, 12].

Hypertrophic cardiomyopathy (HCM) is identified by an increase in left ventricular (LV) wall thickness beyond what can be attributed to abnormal loading conditions, where both septal and free wall thickness surpass two standard deviations from the normal values for weight, age, and body surface area [13].

Diagnosis of restrictive cardiomyopathy (CMP) occurs when there is biatrial enlargement, along with indications of diastolic dysfunction and restrictive ventricular physiology [11, 12].

Diagnosis of noncompaction cardiomyopathy (CM) is established when the ratio between the thin, compacted epicardial layer and the noncompacted endocardial layer exceeds 2 at the end of systole. This condition is characterized by prominent trabeculation and deep recesses that connect with the left ventricular cavity but not with the coronary circulation [11, 12].

Arrhythmogenic cardiomyopathy is identified when there is a suspicion of acquired and progressive dystrophy in the ventricular myocardium, characterized by fibro-fatty replacement. This condition may be linked to diminished cardiac function due to arrhythmias and fibro-fatty myocardial replacement [11, 12].

LV systolic dysfunction is characterized by a left ventricle ejection fraction (LVEF) below 50%, while severe LV systolic dysfunction is designated by an LVEF below 30% [11, 12].

LV dilation is identified when the end-diastolic diameter exceeds 2 Z scores for body surface area [12].

## Results

During the study period, 229 pediatric patients were diagnosed to have cardiomyopathy. Dilated cardiomyopathy (DCM) was the most prevalent type, affecting 160 patients (70% of CMP cases), followed by hypertrophic cardiomyopathy (HCM) in 60 patients (26.2%), isolated left ventricular noncompaction in five patients (2.2%), and restrictive cardiomyopathy (RCM) in four patients (1.7%) (Table 1).

**Table 1:** All cardiomyopathy cases characteristics and outcome cross-tabulated according to the type of cardiomyopathy

		Diagnosis CMP Type				
		DCM	HCM	RCM	LVNC	Total
Gender	Male	80	32	1	4	117
	Female	80	28	3	1	112
Mode of presentation	Heart failure	118	47	4	1	170
	Cardiogenic shock	29	1	0	0	30
	Asymptomatic	13	12	0	4	29
Syndromic	Yes	14	3	0	1	18
	No	146	57	4	4	211
Primary or secondary CMP	Primary	76	27	4	3	110
	Secondary	84	33	0	2	119
Suspected viral illness	Yes	18	0	0	0	18
	No	113	60	4	5	182
	Unknown	29	0	0	0	29
Genetic study	Positive/Abnormal	21	27	0	2	50
	Negative/Normal	39	0	0	0	39
	Unknown	100	33	4	3	140
Admission on first presentation	Yes	112	34	3	0	149
	No	31	26	1	5	63
	Unknown	17	0	0	0	17
Consanguineous parents	Yes	41	22	0	1	64
	No	18	1	0	0	19
	Unknown	101	37	4	4	146
Family history of CMP	Yes	17	0	0	0	
	No	106	1	0	0	
	Unknown	12	0	0	0	
Final results	Static	17	15	3	5	40
	Normalized	57	0	0	0	57
	Improving	29	6	0	0	35
	Expired	48	39	1	0	88
	Lost	9	0	0	0	9
Alive dead	Alive	103	21	3	5	132
	Expired	48	39	1	0	88
	Lost	9	0	0	0	9

The gender distribution was nearly equal (51% males and 49% females). The average age at presentation for all patients was 16.8 months (± standard deviation of 28.7 months), ranging from the first week of life to 13 years of age. Presentation occurred at 6 months or earlier in 118 patients (51.5%) and beyond 6 months in 111 patients (48.5%). The mean weight at presentation was 8.12 kg (± SD of 5.5 kg), with a range from 2 to 43.5 kg.

The primary mode of presentation was through signs and symptoms of heart failure in 170 patients (74.2%). Cardiogenic shock was observed in 30 patients (13.1%), more frequently in those with DCM. Additionally, 29 patients (12.7%) were asymptomatic at the initial presentation.

Parental consanguinity was noted in 90 patients (39.3%) among all patients with cardiomyopathy. Positive consanguinity was observed in 58 of those with DCM (36.3%) and 29 of those with HCM (48.3%).

A significant proportion of patients with cardiomyopathy required hospital admission (69.4%), and 123 patients (53.7%) necessitated ICU admission at the initial presentation. Invasive ventilatory support was needed in 55.5% and inotropic support in 53.3%.

The causes of cardiomyopathy remained undisclosed in most patients with negative family history and genetic metabolic studies. However, the cause was identified in 110 patients (48%), predominantly post-viral myocarditis in DCM cases and genetic/metabolic factors in HCM patients. (Table 1).

A considerable number of individuals with dilated cardiomyopathy (DCM) presented with a history indicative of viral infection, prompting suspicion of viral myocarditis as the underlying cause. The confirmation of genetic and/or metabolic disorders occurred in 49 patients (21.4%), with VLCAD deficiency identified in 16 patients (7%) and an ELAC2 gene defect in ten patients (4.4%) (Tables 2 and 3).

**Table 2:** Secondary cardiomypathy, causes and CMP type

	Diagnosis CMP Type				
Secondary to what?	DCM	HCM	RCM	LVNC	Total
DCM idiopathic	84	0	0	0	84
HCM	2	31	1	0	34
Genetic pending results	21	2	0	0	23
Post-viral myocarditis	18	0	0	0	18
VLCAD	1	14	0	1	16
ELAC2	2	8	0	0	10
Suspected metabolic disorder	8	0	0	0	8
LV noncompaction	2	0	0	3	5
Duchene muscular dystrophy	4	0	0	0	4
Alstrom syndrome	4	0	0	0	4
Noonan syndrome	0	2	0	1	3
RCM	0	0	3	0	3
NEXN gene defect	2	0	0	0	2
WPW	2	0	0	0	2
Prader–Willi Syndrome	2	0	0	0	2
Beckwith-Wiedemann syndrome	0	1	0	0	1
Chronic kidney disease, nephrotic syndrome	1	0	0	0	1
Hypochondroplasia	1	0	0	0	1
GAA	0	1	0	0	1
Pompe disease	0	1	0	0	1
SCA	1	0	0	0	1
Sepsis	1	0	0	0	1
Suprarenal mass and systemic hypertension	1	0	0	0	1
Connective tissue disorder	1	0	0	0	1
Vit. D deficiency	1	0	0	0	1
Anemia	1	0	0	0	1
Autism and DCM M mode 58%	1	0	0	0	1
Total	160	60	4	5	229

Diagnosis CMP-type cross-tabulation

**Table 3:** Outcome of patients versus secondary causes cross-tabulation

	Alive	Expired	Total
Normal	102	47	149
VLCAD	9	7	16
Positive pending	8	16	24
ELAC2	0	10	10
Suspected metabolic disorder	1	7	8
Douchene muscular dystrophy	4	0	4
Alstrom syndrome	4	0	4
Noonan syndrome	3	0	3
NEXN gene mutation	3	0	3
Prader–Willi syndrome	2	0	2
GAA	0	1	1
Beckwith-Wiedemann syndrome	1	0	1
Hypochondroplasia	1	0	1
Connective tissue disorder	1	0	1
Pompe disease	1	0	1
SCA	1	0	1
Total	141	88	229

The overall outcomes for all cardiomyopathy cases included cardiac function normalization in 57 patients (25%), improvement in 44 (19.2%), a static condition in 40 (17.5%), cardiac transplantation in 2, and mortality in 88 patients (38.4%) (Table 4).

**Table 4:** DCM cases, comparison between those who are alive and dead

		Alive	Died	Total	*P*-value	Odds ratio	Relative Risk
Gender	Male	56	24	80	0.568	1	1
	Female	56	24	80			
Mode of presentation	Heart failure	81	37	118	0.038		
	Cardiogenic shock	18	11	29			
	Asymptomatic	13	0	13			
Primary or secondary	Primary	57	19	76	0.127	0.63	0.72
	Secondary	55	29	84			
Syndromic	Yes	9	5	14	0.415	1.33	1.21
	No	103	43	146			
Primary or secondary	Primary	53	19	4	0.483	0.62	0.72
	Secondary	50	29	5			
Suspected viral illness	Yes	22	2	24	0.008	0.18	0.25
	No	90	46	136			
LV noncompaction	Yes	84	46	130	0.001	7.67	5.31
	No	28	2	30			
Consanguineous parents	Yes	32	26	58	0.002	2.95	2.08
	No	80	22	102			
F/H of CMP	Yes	9	12	21	0.005	3.81	2.21
	No	103	36	139			
Genetic study	Positive/Abnormal	17	3	20	0.012		
	Negative/Normal	35	6	41			
	Pending	9	21	30			
	Normal	51	18	69			
Age of presentation	6 months or Less	40	22	62	0.152	1.52	1.34
	More than 6 months	72	26	98			
Ejection fraction at presentation	20% or Less	10	25	35	< 0.001	11.09	3.88
	More than 20%	102	23	125			
Admission on first presentation	Yes	78	44	122	0.002	4.79	3.43
	No	34	4	38			
ICU admission	Yes	51	29	5	0.045	2.27	1.81
	No	40	10	0			
IVIg administration	Yes	35	9	44	0.074	0.51	0.61
	No	77	39	116			
ICU admission	Yes	57	32	89	0.047	1.93	1.6
	No	55	16	71			
Ventilation	Yes	74	21	95	0.007	0.4	0.53
	No	38	27	65			
Inotropes	Yes	70	21	91	0.022	0.47	0.59
	No	42	27	69			
Carvidolol	Yes	10	8	18	0.127	2.04	1.58
	No	102	40	142			
Digoxin	Yes	39	16	55	0.503	1.22	1.14
	No	73	32	105			

Among DCM patients, 35% experienced normalized cardiac function, 11% had a static clinical condition, 18% showed improvement with residual cardiac dysfunction, and 30% died during the follow-up period.

**Fig. 1:**
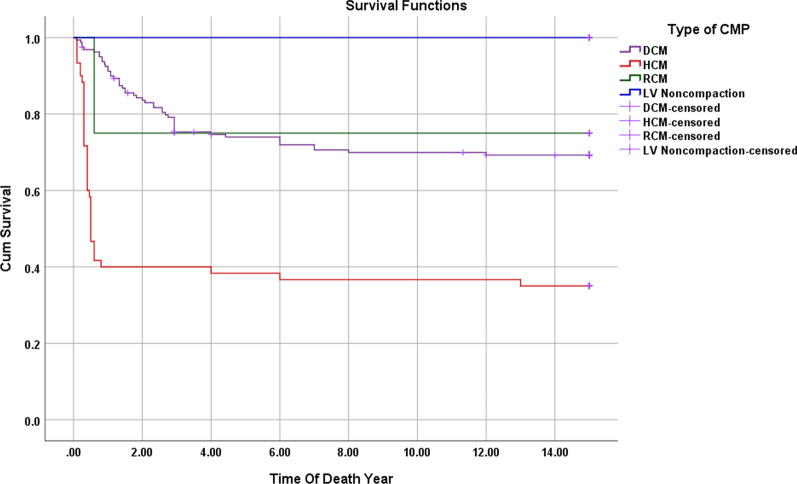
Kaplan–Meier survival curve for all cases divided according to the type of cardiomyopathy. Patients with HCM have a higher mortality rate and die at an earlier age when compared with DCM and other types of CMP. No mortality among patients with isolated LVNC

For hypertrophic cardiomyopathy (HCM) patients, 65% died, predominantly within the first year post-diagnosis. The likelihood of death was higher in those with HCM (especially those diagnosed at 6 months of age or younger) and in patients with dilated cardiomyopathy (DCM) with an ejection fraction (EF) of 20% or less (Fig. 1). Most fatalities occurred during acute presentation or after the first 2 years of diagnosis.

Symptomatic management, including diuretics, ACE inhibitors, and spironolactone, was commonly prescribed for most DCM and restrictive cardiomyopathy (RCM) patients. Two patients only had heart transplantation (one with DCM and one with RCM).

Comparing survivors and deceased patients across all cardiomyopathy cases revealed that deceased individuals had lower weight and height, indicative of a potential failure to thrive due to disease severity. Presentation at less than 6 months carries a risk for mortality for all patients. This was true for HCM patients but was not statistically significant in patients with DCM. The requirement for an ICU admission, the presence of a family history of CMP, and positive consanguinity were risk factors for bad outcomes.

### Dilated CMP

Of 229 patients diagnosed with cardiomyopathy (CMP), 160 (70%) had features characteristics of dilated cardiomyopathy (DCM). The mode of presentation was mainly with signs and symptoms of heart failure (118 patients 74%). Cardiogenic shock was observed in 29 patients (18%). The mean ejection fraction (EF) at presentation was 28 ± 5%. A discernible cause was identified in 76 patients (47.5%), with genetic factors and post-viral infection being the most common probable causes. Genetic testing yielded positive results in 20 patients (12.5%).

During the initial presentation, 70% required hospital admission, and 53% were admitted to the ICU. Intravenous immunoglobulin (IVIg) was administered to 44 patients (27.5%). Subsequent outcomes revealed that 35% normalized, 23.8% showed improvement, 10.6% had a static cardiac function, and 30% succumbed during the follow-up period. One patient underwent cardiac transplantation. The mean time of death after presentation was 12 months ± 10 months.

A comparison between surviving and deceased DCM patients revealed that EF at presentation as well as the rate of improvement in cardiac function with time was a major determinant for possible mortality. The mean EF for survivors and deceased patients was 30 ± 10% and 24 ± 8%, respectively, with a statistically significant difference (*p*-value < 0.001). Patients with an EF less than 20% at presentation faced a mortality risk, with a relative risk of 3.88 and an odds ratio of 11.09 compared to those with an EF of more than 20% at presentation.

**Fig. 2:**
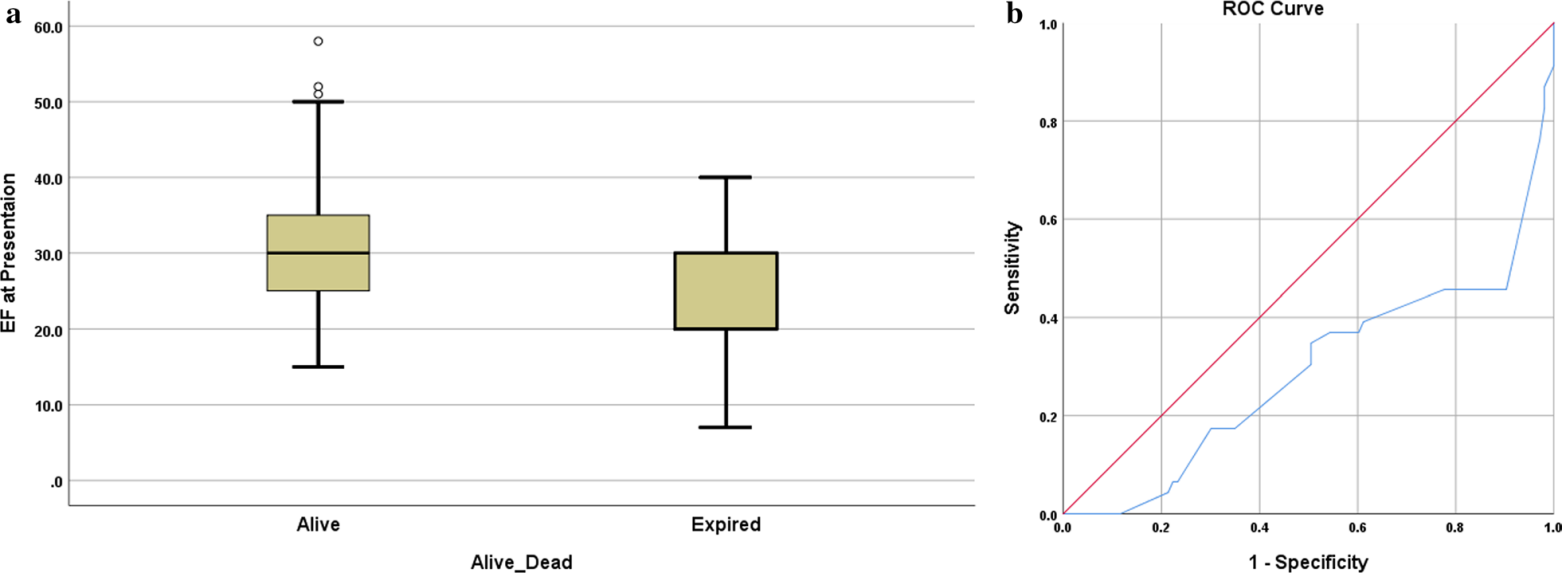
**a** & **b**: Ejection fraction (EF) in patients with DCM. The box and plot graph reveal that dead patients had lower EF at presentation (**a**). The ROC curve (**b**) reveals that EF at presentation of 20% or less carries a high risk for mortality with a sensitivity and specificity of 76% and 97%, respectively, with an area under the curve of 0.323, and a *P*-value of 0.001 (95% CI 0.22-0.427)

**Fig. 3:**
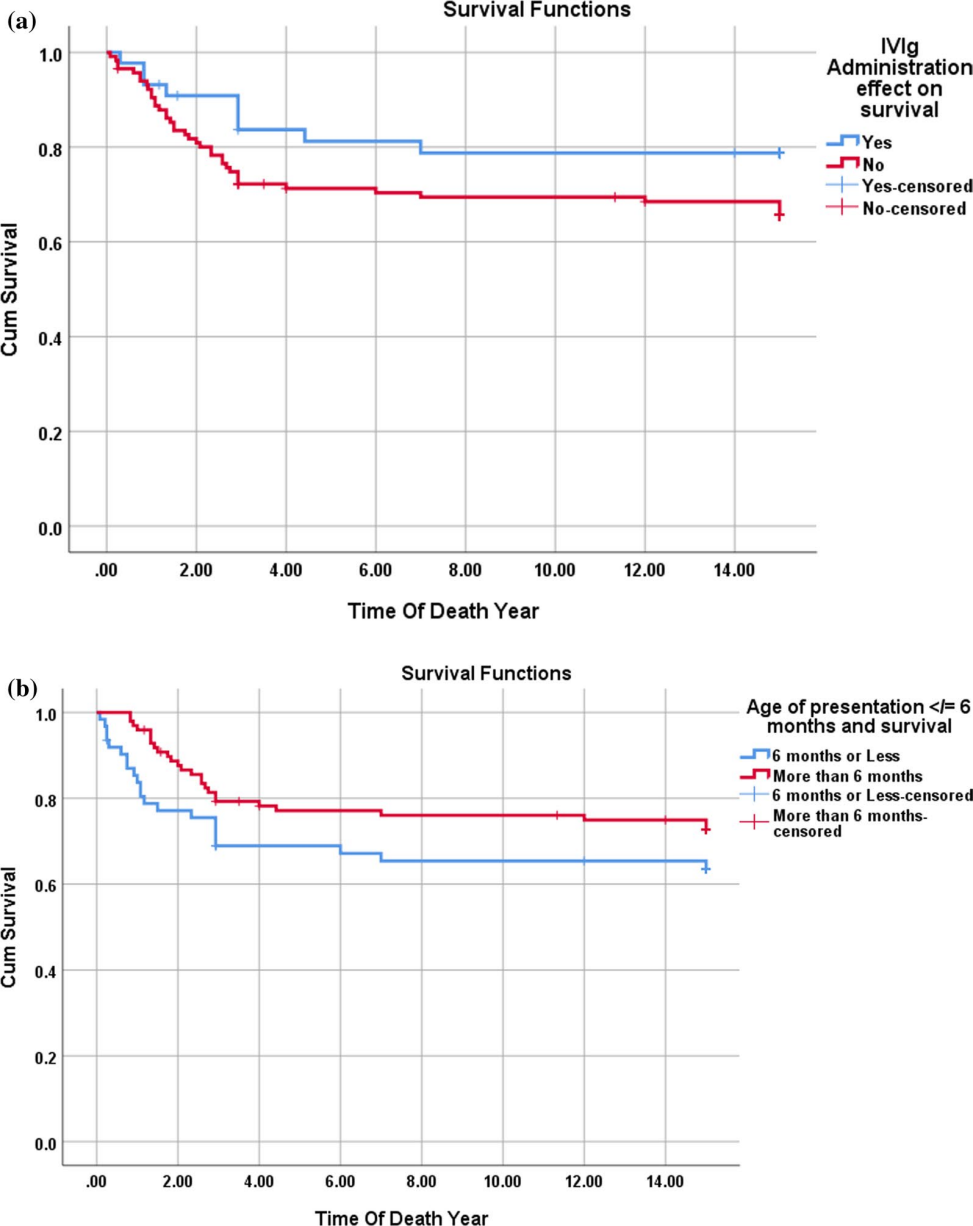

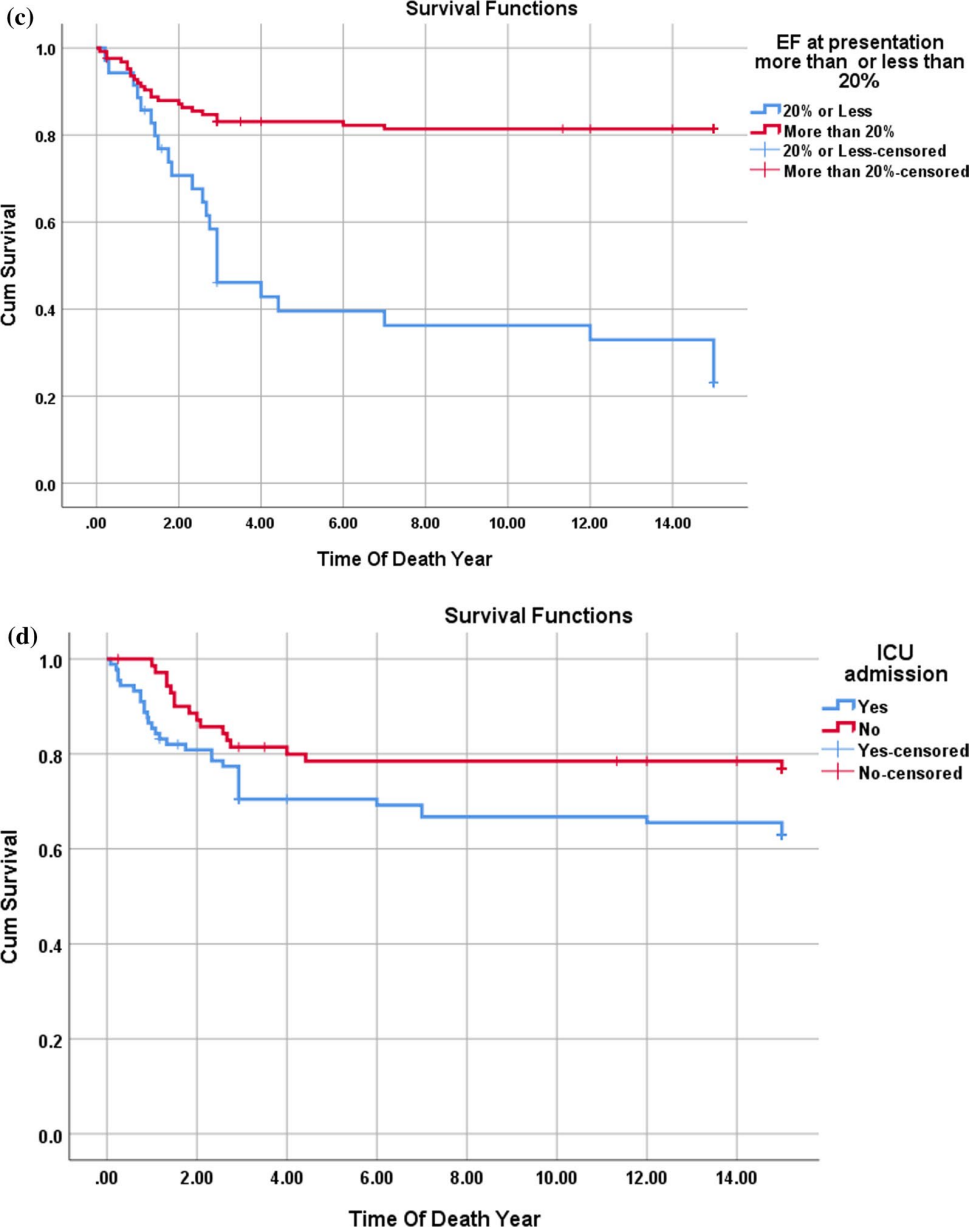
**a**–**d**: Kaplan–Meier survival curve for DCM patients with the multivariate assessment of the effect of IVIg administration (**a**), age of presentation (**b**) EF at presentation (**c**), and ICU admission (**d**) on survival. The mortality rate among DCM patients was 30% with most deaths occurring in the first 2 years after the diagnosis. IVIg administration had no significant effect on reducing the mortality (*p*-value 0.074). EF at the presentation of 20% or less had a significant risk for a bad outcome (*p*-value < 0.001). Patients admitted to the ICU had a higher mortality rate (*p*-value 0.047)

There was a change in the mortality among those who received IVIg during the initial illness with nine mortalities among the 44 patients who received IVIg and 39 mortalities among the 87 patients who did not, showing a p-value of 0.074 (RR 0.61 and odds ratio of 0.51). On the initial presentation, patients admitted to the ICU, ventilated patients, and those requiring inotropic support exhibited lower mortality rates than other groups (*p*-value 0.001) (Table 5 and Figs. 2 and 3).

**Table 5:** HCM cases comparison between those who are alive and dead

		Alive	Dead	*P*-value	Odds ratio	Relative Risk
Gender	Male	13	19	0.241	0.58	0.83
	Female	8	20			
Admission On first presentation	Yes	3	31	0.001	23.25	2.96
	No	18	8			
Mode Of presentation	Heart failure	9	38	0.001		
	Cardiogenic shock	0	1			
	Asymptomatic	12	0			
Primary or secondary	Primary	6	21	0.053	2.92	1.43
	Secondary	15	18			
ICU admission	Yes	5	28	0.001	8.15	2.08
	No	16	11			
Syndromic	Yes	3	0	0.039	0	0
	No	18	39			
Ventilation	Yes	21	3	0.001	0	0.13
	No	0	36			
Inotropes	Yes	21	2	0.001	0	0.09
	No	0	37			
Consanguineous parents	Yes	14	15	0.674	0.31	0.67
	No	7	24			
Family history Of CMP	Yes	10	15	0.339	0.69	0.88
	No	11	24			
Genetic study	Positive/Abnormal	12	15	0.270	0.51	0.78
	Pending	0	2			
	Normal	9	22			
Age of presentation less than 6mo	6 months or Less	17	37	0.106	4.35	2.06
	More than 6 months	4	2

### Hypertrophic CMP

Out of the 229 pediatric patients diagnosed with cardiomyopathy (CMP), 60 patients (26%) exhibited characteristics of hypertrophic cardiomyopathy (HCM). Notably, individuals with HCM presented at a younger age compared to those with dilated cardiomyopathy (DCM).

The mean age ± SD at presentation was 6.7 months ± 20.2 months, and the mean weight was 5.4 kg ± 3.8 kg. Hospital admission was required for 34 patients at the initial diagnosis (57%), with 33 patients (55%) requiring ICU admission. Among HCM patients, 25 individuals (42%) had a family history of cardiomyopathy.

A discernible cause for HCM was identified in 29 patients (48.3%), predominantly of genetic and/or metabolic origin. Genetic testing yielded positive results in 28 patients (47%), with VLCADD identified in 14 patients (23.3%) and an ELAC2 gene defect in eight patients (13.3%) (Tables 2 and 3).

The outcomes for patients with HCM were as follows: 10% showed some improvement, 25% had a static cardiac condition and function, and 65% died during the follow-up period. The mean time of death after presentation was 9 months ± 4 months.

**Fig. 4:**
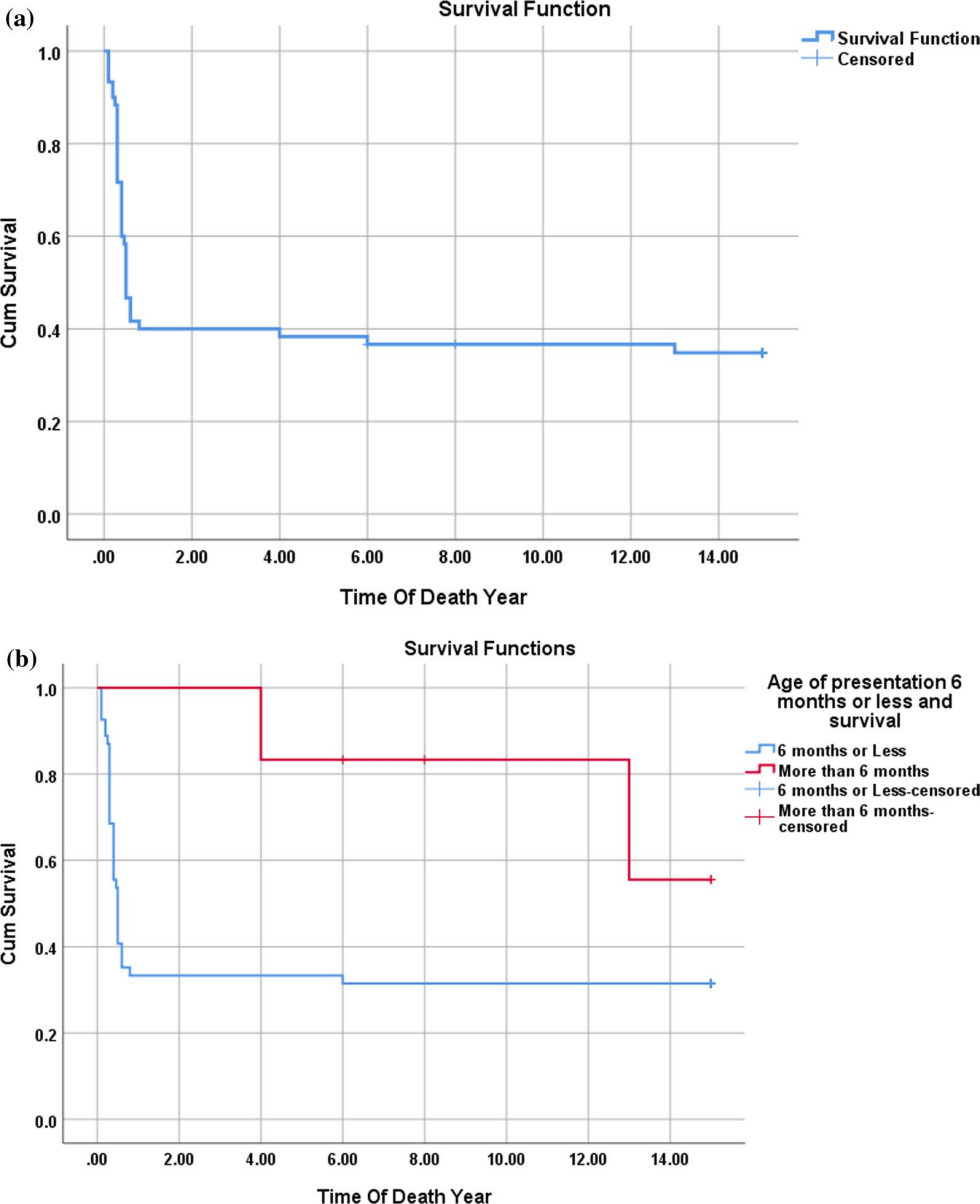

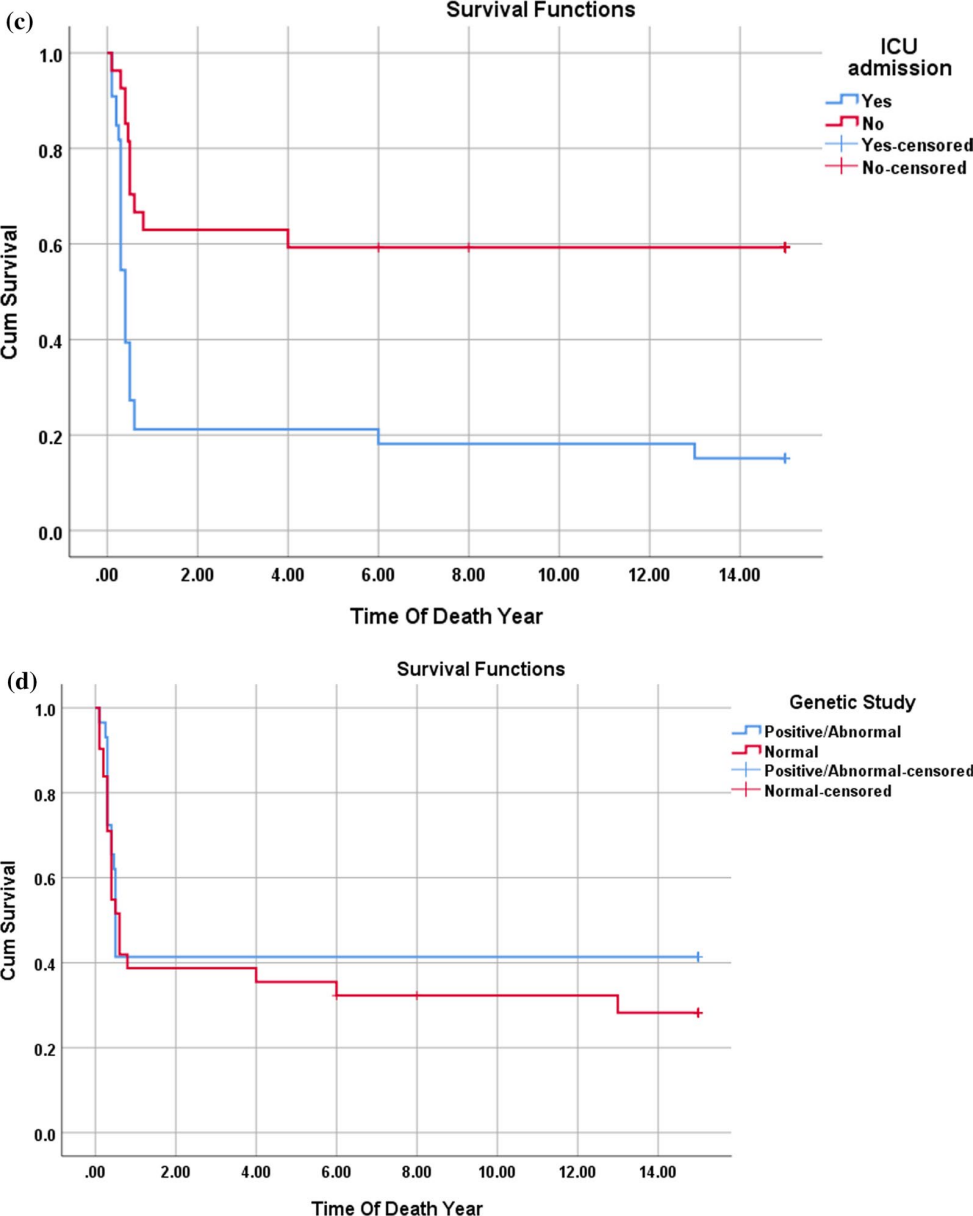
**a**–**d**: Kaplan–Meier survival curve for HCM patients (**a**) with the multivariate assessment of the effect of the age at presentation (**b**), ICU admission (**c**), and results of the genetic study (**d**) on survival. The mortality rate among patients with HCM was 65%, (35 patients died), most of them in the same year of the diagnosis (First year of life). Presentation with HCM at 6 months or less and/or ICU admission was risk for mortality

Comparing HCM patients who survived with those who did not reveal that presentation at the age of 6 months or less, along with the presence of abnormal genetic investigations, significantly increased the risk of mortality. Presentation at 6 months or less carried a risk for mortality, with a relative risk of 1.9 and an odds ratio of 3.3.Additionally, the need for ICU admission during the initial presentation of HCM patients emerged as a risk factor for mortality within our cohort (Table 5, Fig. 4).

### Restrictive CMP

In this study, four patients (1.7%) exhibited characteristics of RCM. The average age at presentation was 18 months ± 16 months. All presented with signs and symptoms of heart failure. One of them died at the age of 2 years, and the others are static on medications for heart failure.

### Isolated LV noncompaction

In this study, five patients (2.2%) had features of isolated LVNC. All are alive. One patient has features of Noonan syndrome, and one had a positive study for VLCAD.

## Discussion

Cardiomyopathy (CMP) in children has a diverse clinical as well as genetic characteristics. It is associated with significant morbidity and mortality. The precise incidence remains uncertain, with reported annual rates ranging between 0.65 and 1.24 per 100,000 children. Dilated cardiomyopathy (DCM) and hypertrophic cardiomyopathy (HCM) are the predominant types, accounting for 50 to 58% and 25 to 42%, respectively [2, 14].

The accurate incidence in our society remains uncertain due to inadequate registration of patients with cardiomyopathy (CMP) and the existence of undiagnosed cases. PSCC-Qassim is recognized as the sole referral cardiac center in the region, catering to a population of approximately 1.2 million. In our study, 70% of our patients were diagnosed with dilated cardiomyopathy (DCM), while 26% had hypertrophic cardiomyopathy (HCM).

Both HCM and DCM exhibit a familial pattern, marked by notable genetic heterogeneity. The predominant inheritance pattern is autosomal dominant, although autosomal recessive, X-linked, and mitochondrial inheritance patterns have also been documented [10, 15]. Many patients in our cohort had consanguineous parents and a positive family history of cardiomyopathy, particularly prevalent in those with hypertrophic cardiomyopathy (HCM).

Disease-associated mutations have been identified in over 50 genes, with some overlap between categories. Genetic causes are more prevalent in familial HCM, accounting for up to 65% of patients, and less frequently in other types of cardiomyopathies (e.g., 50% in arrhythmogenic right ventricular dysplasia, and 30% in dilated cardiomyopathy). To address the significant genetic heterogeneity and uncover novel genes, we employed whole-exome sequencing (WES) as a primary diagnostic test in our patients, particularly those with HCM, following the approach adopted by other researchers in the study of cardiomyopathy [10, 16].

In our cohort, the most prevalent genetic abnormalities identified were ELAC2 mutations and very long-chain acyl-CoA dehydrogenase deficiency (VLCADD). Within our cohort, all patients with ELAC2 mutations and 44% of patients with VLCADD experienced mortality. Notably, it has been reported that nearly one-third of patients with ELAC2 mutations succumb during the initial presentation [17].

According to data from the Pediatric Cardiomyopathy Registry published in 2003, about one-third of children diagnosed with cardiomyopathy had a confirmed cause. The documented causes encompassed myocarditis (16% in dilated cardiomyopathy—DCM), metabolic factors (4% in DCM, 9% in hypertrophic cardiomyopathy—HCM), syndromic conditions (1% in DCM, 9% in HCM), and neuromuscular causes (9% in DCM, 9% in HCM) [18].

Infants and children diagnosed with heart failure are currently assessed using the Ross heart failure class, which grades symptoms related to respiration, feeding, and weight gain. Although widely adopted, it is important to note that this classification lacks formal validation against outcomes. Moreover, patients with hypertrophic cardiomyopathy (HCM), restrictive cardiomyopathy (RCM), and noncompaction cardiomyopathy (NCM) may display signs of heart failure with preserved ejection fraction [19].

Echocardiography serves as the primary diagnostic tool for establishing the diagnosis and cardiomyopathy (CMP) phenotype. Following the diagnosis, serial echocardiography is conducted during the acute setting and in the follow-up period. In children with dilated cardiomyopathy (DCM), the extent of ventricular dilation and dysfunction stands as a robust predictor of mortality or transplantation. For patients with hypertrophic cardiomyopathy (HCM), the thickness of the septal wall has been linked to the risk of sudden death [19, 20].

Cardiac MRI proves valuable in identifying inflammatory processes, aiding in the definition of the underlying cause and contributing to the management and risk stratification of cardiac conditions [6].

In dilated cardiomyopathy (DCM), children often exhibit elevated cardiac biomarkers, including B-type natriuretic peptides [21].

### Dilated cardiomyopathy

Dilated cardiomyopathy (DCM) comprises two-thirds of cardiomyopathy cases in our cohort. DCM serves as the ultimate common pathway for various pathological processes that result in left ventricular (LV) dilation and systolic dysfunction, often presenting with signs and symptoms of congestive heart failure. In roughly two-thirds of patients, the underlying cause of DCM remains idiopathic. Potential contributors to DCM include antecedent viral myocarditis, generalized myopathies, and abnormalities in sarcomeric, sarcolemmal, cytoskeletal, or nuclear proteins [22].

Due to the associated risks with endomyocardial biopsy (EMB), the initial diagnosis of myocarditis typically relies on clinical presentation. Moreover, EMB is not widely available and is not routinely performed [23]. Globally, similar to our society, the true prevalence of pediatric myocarditis remains unknown. Myocarditis is reported to contribute to 17% of sudden cardiac deaths in children younger than 16 years old [14]. In our cohort, none of the individuals underwent endomyocardial biopsy (EMB) or cardiac MRI.

The diagnosis of myocarditis was established based on a history of viral infection along with clinical and laboratory evidence of myocardial cell injury. Although this represents a limitation in our study, the focus remains on potential genetic causes and the outcomes associated with various types of cardiomyopathy. It is worth noting that EMB is not recommended for infants weighing less than 10 kg or for hemodynamically unstable patients. The general recommendation is to perform EMB only if confirming the clinical diagnosis of myocarditis would have a clear impact on the patient’s treatment plan, such as listing for transplantation [24].

Cardiac MRI has emerged as the preferred noninvasive test for diagnosing myocarditis. The MRI Lake Louise Criteria (LLC) diagnostic criteria for myocarditis rely on the identification of tissue edema, hyperemia, and necrosis, forming the foundational features observed in both acute and chronic stages [6].

The clinical presentation of children with dilated cardiomyopathy (DCM) varies from asymptomatic cases to acute decompensated heart failure and cardiogenic shock. According to literature, a considerable number of children with DCM necessitate hospitalization, with 54% receiving inotropic support, 41% requiring mechanical ventilation, 13% needing extracorporeal membrane oxygenation (ECMO) support, and 11% undergoing urgent transplantation [25, 26]. In our cohort, 70% of our patients needed hospitalization, with 54% requiring admission to the intensive care unit (ICU) at the initial presentation. Notably, none of the patients in our cohort underwent extracorporeal membrane oxygenation (ECMO) support.

The rate of complete spontaneous recovery in pediatric myocarditis/dilated cardiomyopathy (DCM) following disease onset ranges from 22 to 37% of patients during the follow-up period [4, 27]. Notably, almost 35% of our patients with DCM experienced normalized cardiac function.

The risk factors for death and transplantation in children varied depending on the etiology of dilated cardiomyopathy (DCM). Some reports indicate that predictors of a poor outcome include an initial left ventricular ejection fraction (LVEF) less than 30%, age less than 2 years at presentation, a prolonged course of the disease, and a high level of N-terminal pro-brain natriuretic peptide [27]. On the other hand, younger age at presentation and a lower left ventricular end-diastolic dimension (LVEDD) z-score were reported as independent predictors of normalization [4]. In comparison with idiopathic dilated cardiomyopathy (IDCM), familial dilated cardiomyopathy (FDCM) was reported to have a lower cumulative incidence of death (*P* = 0.04; hazard ratio 0.64, *P* = 0.06), with no significant difference in the risk of transplantation or the combined death or transplant outcome [22, 28].

In this descriptive study, results of multivariate survival analysis for patients with dilated cardiomyopathy (DCM) reveal that most deaths occur in the first 2 years after diagnosis. Risk factors for adverse outcomes include an ejection fraction (EF) at presentation of 20% or less (*p*-value < 0.001), admission to the hospital (and intensive care unit (ICU)) at the first presentation, consanguineous parents, and a positive family history of cardiomyopathy. Patients who received inotropic medications had a lower mortality rate (P-value 0.022). Additionally, patients with DCM and positive genetic studies, except for ELAC2 mutation, have a better prognosis than those with unknown/idiopathic DCM.

However, organ shortage poses a risk, particularly for children on waiting lists. Furthermore, a strong and reliable social support system is crucial for the long-term success of HTx [29]. In addition to the lifelong use of immunosuppressant, acute graft rejection, and chronic graft failure are important challenges with HTx. In our cohort, only two patients underwent heart transplantation. The long-term survival rate of children with cardiomyopathy after HTX in experienced centers is high. Morbidity and mortality were higher in patients with systemic diseases than in those with cardiac-specific conditions [30].

The effectiveness of Beta-Blockade therapy, such as Carvedilol, metoprolol, or bisoprolol, in children, including those with a structurally normal heart, remains unclear. These medications may be initiated in the treatment of moderate to severe systolic dysfunction of a systemic left ventricle.

Intravenous immunoglobulin G (IVIG) is not recommended as a routine treatment for myocarditis. Although some case reports and retrospective studies have suggested a potential benefit of IVIG in the treatment of myocarditis, there is a lack of higher-quality studies with low bias risk and larger sample sizes to support its routine use [31]. In our study, IVIG administration showed no significant effect in reducing mortalities (*p*-value 0.074).

Corticosteroids are not recommended as a routine treatment for myocarditis, particularly in the absence of robust randomized controlled trial evidence.

### Hypertrophic cardiomyopathy

More than 1400 mutations in various sarcomeric genes have been identified in approximately 70% of patients with hypertrophic cardiomyopathy (HCM) [32]. Infants with hypertrophic cardiomyopathy (HCM) stemming from malformation syndromes and inborn errors of metabolism commonly exhibit symptoms in infancy and, often linked to neurological and musculoskeletal issues. The mortality rate for these infants is notably elevated, reaching 30% within a two-year period, surpassing that of older children. Poor prognostic factors include mixed phenotypes, presentation at 1 year of age or younger, low weight, the presence of signs and symptoms of heart failure, lower left ventricular fractional shortening, and increased left ventricular posterior wall septal thickness at the time of diagnosis [33].

Patients with the infantile form of Pompe disease might respond to enzyme replacement therapy, which can be effective in reducing the progression of left ventricular hypertrophy if initiated early. Enzyme replacement therapy or bone marrow transplantation is currently the treatment of choice in some patients with specific types of lysosomal storage diseases, namely Mucopolysaccharidoses I, II, IV, and VI [34, 35].

For patients with restrictive cardiomyopathy (RCM), the 5-year mortality rate is high and has been reported to be 68% from the time of diagnosis [2].

At least 5% of children diagnosed with cardiomyopathy have some form of left ventricular noncompaction (LVNC). This condition may occur in isolation (23%) or be associated with dilated or hypertrophic cardiomyopathy (59% and 11%, respectively). There is a tendency for both under- and over-diagnosis of LVNC, possibly stemming from the lack of consensus on diagnostic criteria for LVNC [19].

Noncompaction of the left ventricle is a common finding in Barth syndrome, which is an X-linked recessive disorder caused by a mutation in the tafazzin (TAZ) gene on chromosome Xq28 [36]. LVNC can also be associated with other inborn errors of metabolism and certain congenital heart diseases, including septal defects and pulmonary stenosis [36].

Children with noncompaction cardiomyopathy (NCM) who have normal cardiac dimensions and systolic function are at a very low risk for adverse outcomes. However, those with NCM mixed with other phenotypes, such as dilated cardiomyopathy (DCM) or hypertrophic cardiomyopathy (HCM), have a worse prognosis with an increased incidence of death or transplantation (18–25%) [19].

## Conclusions

Cardiomyopathy constitutes a serious and heterogeneous group of myocardial disorders with multifactorial etiologies. It is a frequent cause of heart failure and the most common reason for heart transplantation in children older than 1 year. The majority of children are diagnosed with dilated cardiomyopathy (DCM) or hypertrophic cardiomyopathy (HCM). Diagnosis confirmation relies on echocardiographic measurements and assessment of ventricular function. The mortality for cardiomyopathy in children can reach up to 40%, with a mortality rate of 30% for patients with DCM and 65% for those with HCM. Predictors of poor prognosis (death and/or heart transplantation) vary depending on the type of cardiomyopathy. Patients with HCM presenting in the first year of life and those with DCM with an ejection fraction at presentation of 20% or less have a poor prognosis. The presence of abnormal genetic abnormalities carries a poorer prognosis in HCM compared to DCM patients. Genetic study using whole-exome or whole-genome sequencing is recommended for all patients diagnosed with cardiomyopathy and at-risk family members.

### Limitations of the study

1. Retrospective nature of the study.
2. The use of clinical diagnoses or suspicion of myocarditis, with no endomyocardial biopsy.
3. Limited use of MRI for initial diagnosis.
4. The limited use of brain natriuretic peptide (BNP) test for assessment and follow-up of patients with heart failure.
5. The low number of patients with heart transplantation because our center does not have a heart transplant program.
6. The absence of ECMO support.
7. The absence of a clear hospital policy regarding the use of immunomodulatory therapy for patients with suspected myocarditis.
8. In the study, no genetic result available after taking samples for investigation was considered as negative genetic testing after contacting the genetic test center.

## Abbreviations

AICD: Automatic implantable cardioverter -defibrillator
ALCAPA: Anomalous left coronary artery from the pulmonary artery
ARVD/C: Arrhythmogenic right ventricular dysplasia/cardiomyopathy
AS: Aortic stenosis
BNP: brain natriuretic peptide
CMP: Cardiomyopathy
CMRI: Cardiac magnetic resonance imaging (CMRI)
CoA: Coarctation of the aorta
DCM: Dilated cardiomyopathy
ECMO: Extracorporeal membrane oxygenation
ELAC2 gene: ElaC Ribonuclease Z 2 gene
EMB: Endomyocardial Biopsy
HCM: Hypertrophic cardiomyopathy
HTx: Heart transplantation
ICU: Intensive care unit
IVIG: Intravenous immunoglobulin
LLC: Lake Louise criteria
LV: Left ventricular
LVEDD: Left ventricular end diastolic dimension
LVEF: Left ventricular ejection fraction
LVNC: Left ventricular noncompaction
NCM: Noncompaction cardiomyopathy
RCM: Restrictive cardiomyopathy
TGT: Targeted genetic test
VLCADD: Very long-chain Acyl-CoA dehydrogenase deficiency
WES: Whole Exome sequencing
WGS: Whole-genome sequencing
